# Medication-Related Hospital Readmissions Within 30 Days of Discharge: Prevalence, Preventability, Type of Medication Errors and Risk Factors

**DOI:** 10.3389/fphar.2021.567424

**Published:** 2021-04-13

**Authors:** Elien B. Uitvlugt, Marjo J. A. Janssen, Carl E. H. Siegert, Eva L. Kneepkens, Bart J. F. van den Bemt, Patricia M. L. A. van den Bemt, Fatma Karapinar-Çarkit

**Affiliations:** ^1^ Onze Lieve Vrouwe Gasthuis OLVG, Department of Hospital Pharmacy, Amsterdam, Netherlands; ^2^ Onze Lieve Vrouwe Gasthuis OLVG, Department of Internal Medicine, Amsterdam, Netherlands; ^3^ Department of Pharmacy, Sint Maartenskliniek, Nijmegen, Netherlands; ^4^ Department of Pharmacy, Radboud University Medical Centre, Nijmegen, Netherlands; ^5^ Department of Clinical Pharmacy and Pharmacology, University Medical Center Groningen, Groningen, Netherlands

**Keywords:** readmission, ADEs, quality of care, medication related problem, transitions in care

## Abstract

**Background:** Hospital readmission rates are increasingly used as a measure of healthcare quality. Medicines are the most common therapeutic intervention but estimating the contribution of adverse drug events as a cause of readmissions is difficult.

**Objectives:** To assess the prevalence and preventability of medication-related readmissions within 30 days after hospital discharge and to describe the risk factors, type of medication errors and types of medication involved in these preventable readmissions.

**Design:** A cross-sectional observational study.

**Setting:** The study took place across the cardiology, gastroenterology, internal medicine, neurology, psychiatry, pulmonology and general surgery departments in the OLVG teaching hospital, Netherlands.

**Participants:** Patients with an unplanned readmission within 30 days after discharge from an earlier hospitalization (index hospitalization: IH) were reviewed.

**Measurements:** The prevalence and preventability of medication-related readmissions were assessed by residents in multidisciplinary meetings. A senior internist and hospital pharmacist reassessed the prevalence and preventability of identified cases. Generalized estimating equation with logistic regression was performed to identify risk factors of potentially preventable medication-related readmissions.

**Results:** Of 1,111 included readmissions, 181 (16%) were medication-related, of which 72 (40%) were potentially preventable. The number of medication changes at IH (Adjusted odds ratio [OR_adj_]: 1.14; 95% CI: 1.05–1.24) and having ≥3 hospitalizations 6 months before IH (ORadj: 2.11; 95% CI: 1.12–3.98) were risk factors of a preventable medication-related readmission. Of these preventable readmissions, 35% were due to prescribing errors, 35% by non-adherence and 30% by transition errors. Medications most frequently involved were diuretics and antidiabetics.

**Conclusion:** This study shows that 16% of readmissions are medication-related, of which 40% are potentially preventable. If the results are confirmed in larger multicentre studies, this may indicate that more attention should be paid to medication-related harm in order to lower the overall readmission rates.

## Introduction

Unplanned hospital readmissions within 30 days are increasingly used as a measure of healthcare quality. Previous studies show that approximately 20% of patients discharged from hospital are readmitted within 30 days of discharge (Jencks et al., 2009) and 5–79% of those readmissions are estimated to be preventable (median: 27%) (Van Walraven et al., 2011). Medication, and more specifically polypharmacy, seems to be one of the causes for these readmissions (Garcia-Caballos et al., 2010; Ahmad et al., 2014). It is estimated that 21% (range: 3–64%) of readmissions are due to medication and a median of 69% (range: 5–87%) of these readmissions were deemed preventable (El Morabet et al., 2018). However, previous studies on the impact of medication on (preventable) hospital readmissions have some methodological flaws (El Morabet et al., 2018). The wide range of point estimates found in previous studies may be due to the small sample size of the reviewed studies, the inclusion of only one department and the method to assess preventability (i.e., by either a pharmacist or physician, not both). Consequently, it is difficult to state how often medication-related readmissions occur. Only six studies have determined the preventability of medication-related readmissions and in only two of these was the preventability assessed by a multidisciplinary method (Ruiz et al., 2008; Rothwell et al., 2011), despite the fact that a multidisciplinary review is recommended (van Doormaal et al., 2008). Furthermore, the type of medication errors involved in preventable medication-related readmissions were unclear and there is a focus on prescribing errors, whereas other medication errors may also be important, such as non-adherence (El Morabet et al., 2018; Parekh et al., 2018). Thus, a clear understanding of the impact and risk factors for adverse drug events on readmissions is lacking. In order to develop interventions to lower overall readmission rates in hospitals it is important to understand the role that medication plays in patient readmissions because medication is the most common therapeutic intervention (World Health Organisation, 2019). Therefore, this study aims to assess the prevalence and preventability of medication-related readmissions within 30 days of discharge, Additionally, potential risk factors associated with preventable medication-related readmissions, the types of medication errors and the medications involved in those readmissions are assessed.

## Methods

### Study Design and Participants

The data for this study were collected within the context of a larger study on all-cause readmissions (van der Does et al., 2020). The all-cause study and the current study used the same inclusion criteria. The all-cause study assessed the extent to which the provided care during an earlier hospitalization, and the subsequent outpatient follow-up care provided by the hospital, was responsible for the readmission. The current study focused on readmissions due to adverse drug events, which are any injuries resulting from medication use, including physical harm, mental harm, or loss of function (El Morabet et al., 2018, see for definitions Supplementary Material). Adverse drug events can result from (non-preventable) adverse drug reactions (defined as a response to a medicine that is noxious and unintended and occurs at doses normally used in man) (World Health Organization, 2006) or (preventable) medication errors (defined as errors in the process of prescribing, dispensing or administering medication that may cause or lead to inappropriate medication use or harm while the medication is in the control of the healthcare professional, patient or consumer) (van den Bemt and Egberts 2007).

A cross-sectional single-centre observational study was conducted from July 15, 2016 until February 28, 2018 at seven clinical departments in the OLVG teaching hospital in Amsterdam, Netherlands.

All seven clinical departments and the hospital pharmacy assigned a resident to review readmissions. All residents received a group training prior to the start of the study regarding the assessment whether the readmission was caused by healthcare (i.e., causality) and the preventability of readmissions (van der Does et al., 2020). In case of replacement of a resident, the new resident received the same training.

All the reviewers had full access to the hospital information system, including laboratory values, medication prescriptions and all notes (i.e., from nurses, physicians, pharmacy, etc.).

Inclusion criteria were: unplanned readmissions of adult patients (≥18 years) within 30 days after discharge from an earlier hospitalization (index hospitalization: IH) to one of the participating departments (cardiology, gastroenterology, internal medicine, neurology, psychiatry, pulmonology and general surgery). Those wards were chosen based on the highest readmission rates in previous years.

**FIGURE 1:**
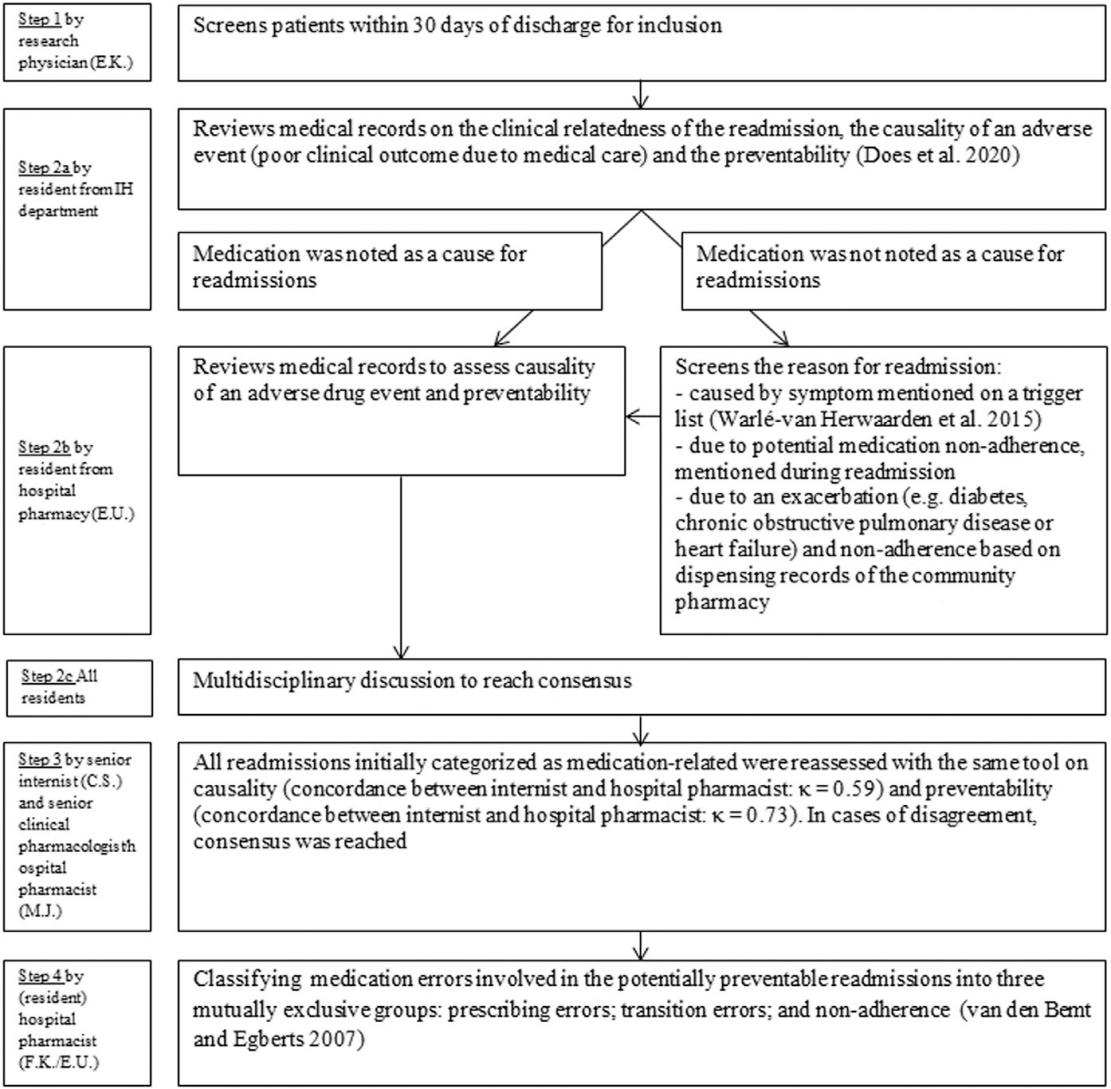
Assessment of causality, preventability and type of medication errors. An adjusted version of the algorithm by Kramer et al. and a modified version of the algorithm by Schumock and Thornton were used to assess the association between the clinical presentation at readmission and the suspected medication (causality) and preventability (Kramer et al., 1979; Schumock and Thornton. 1992; McDonnell and Jacobs. 2002; Lau et al., 2003; Leendertse et al., 2008). Therapy adherence was defined as a refill rate higher than 0.8. The refill rate was defined as the number of daily doses dispensed (refill interval) divided by the total number of days between the first and last prescription in this period (we used a period of 8 months before readmission). If the refill rate could not be calculated but the doctor mentioned the patient as being therapy non-adherent, the patient was classified as such. Likewise, a refill rate above 0.8 can be overruled by a doctor classifying the patient as non-adherent.

Exclusion criteria were: patients who were transferred to another hospital during IH, patients who left the hospital against medical advice during IH and if the readmission was due to attempted suicide. Furthermore, a readmission was excluded if it was deemed unrelated to the IH. This was initially assessed by the study coordinator (a medical doctor) and then double-checked by the resident of the department of IH. Any non-agreement between the two led to the case being discussed at a multidisciplinary meeting (see Figure 1).

The study was approved by the local review board of the hospital (Advies Commissie Wetenschappelijk Onderzoek Medische Ethische Commissie, ACWO-MEC; registration number 16–028). Patient data were obtained and handled in accordance with privacy regulations.

### Usual Care During Index Hospitalization

In the OLVG hospital, medication monitoring is performed by hospital pharmacists using a computerized system to check for the right dose and medication interactions. A Transitional Pharmaceutical Care (TPC) program (Karapinar-Çarkit et al., 2012) was implemented gradually during the study.

• On departments with this TPC program, hospital pharmacy teams performed medication reconciliation upon hospital admission and discharge using the dispensing history of the community pharmacy and information from the patient/carer. Any discrepancies between a patient's actual medication use and the medication prescribed in hospital were discussed with the resident. No formal medication review was performed. However, obvious errors in pharmacotherapy were eliminated: for example, lack of a laxative when an opioid is prescribed or no indication for hypnotics upon discharge and/or addressing a stop date for antibiotics or opioids. The reason for medication changes was explained to the patient during discharge counseling and a written medication summary was provided. The pharmacy team compiled a TPC medication overview that the resident could upload into the discharge letter to the general practitioner.
• On departments where the TPC program was not yet implemented, residents and nurses were responsible for assessing the patient's actual medication use by interviewing patients/carers. If regarded as necessary, they could request the hospital pharmacy to obtain a dispensing history from the community pharmacy. At hospital discharge, the resident uploads information from the hospital's prescribing system or types information into the discharge letter to the general practitioner.

### Assessment of Causality, Preventability and Type of Medication Errors

Figure 1 shows the different steps in the assessment of causality, preventability and type of medication errors.

If medication was noted as a cause for readmissions by the residents, the readmission was included for a review by the resident of the hospital pharmacy. The resident of the hospital pharmacy assessed the causal relationship between the suspected medicine and the reason for readmission (i.e., causality), using an adjusted version of the algorithm of Kramer et al. (1979), see Supplementary Material). This algorithm has been used in a previous study to assess the causality of medication related admissions (HARM-study: Leendertse et al., 2008, see Supplementary Material). This algorithm included three questions: whether the reason for admission is known to be an effect of the suspected medicine, whether alternative causes can explain the relationship between the suspected medicine and the readmission, and whether a plausible time relationship exists between the readmission and the start of medication administration (or the occurrence of the medication error). The subscores of the three questions were added to a total score, and classified as: possible, probable or unlikely causal. Readmissions classified as possible or probable causal were classified as medication related and the preventability of those readmissions was assessed by the same pharmacist according to a modified version of the algorithm by Schumock and Thornton (Schumock and Thornton, 1992; Lau et al., 2003; Leendertse et al., 2008, see Supplementary Material). The algorithm assessed dosing or therapeutic errors (e.g., type of medication inappropriate for the patient’s clinical condition, inappropriate dose or frequency, medication contraindicated), inadequate monitoring of the therapy (e.g., therapeutic drug monitoring or other monitoring), lack of preventative measure (e.g., no laxative with opioid use) or incorrect medication use (e.g., non-adherence). A readmission was considered potentially preventable when a medication error caused the readmission (see Supplementary Material). All potentially preventable readmissions were discussed in multidisciplinary meetings with all residents to reach consensus.

Hereafter all medication-related readmissions were reassessed by a senior internist and senior clinical pharmacologist/hospital pharmacist. The agreement between internist and hospital pharmacist for medication-relatedness’s was *κ* = 0.59 (moderate) and for preventability *κ* = 0.73 (substantial). In case of disagreement, consensus was reached.

Finally, medication errors involved in potentially preventable readmissions were classified by the resident of the hospital pharmacy and the principal investigator/hospital pharmacist into three mutually exclusive groups to determine where in the medication process the error has occurred. These groups included: prescribing errors (defined as dosing/therapeutic errors or inadequate monitoring); transition errors (insufficient communication to the patient and/or next healthcare provider, e.g., regarding a medication change); and non-adherence (medication not used as prescribed by the patient), according to the van den Bemt and Egberts classification (van den Bemt and Egberts, 2007).

### Data Collection

Relevant information on included readmissions was collected from medical records and entered into a data processing program (*Castor* EDC). Potential risk factors were collected based on previous studies (Van Walraven et al., 2011; Feigenbaum et al., 2012; van der Does et al., 2020) (Table 1).

**TABLE 1:** Patient- and index hospitalization-related parameters and adjusted odds ratios and 95% confidence intervals from parameters significantly associated in the univariate analyses.

	Medication-related and potentially preventable N = 72	Not medication-related N = 930	OR (95% CI)	*p*-value	OR_adj_ (95% CI)	*p*-value
**Patient related** ^a^						
Male, n (%)	38 (52.8)	452 (48.6)	1.06 (0.53–2.14)	0.86		
Age in years, mean (SD)	69.5 (13.7)	63.4 (17.4)	1.02 (1.01–1.04)	0.01	1.02 (0.99–1.04)	0.15
Language barrier present, n (%)	25 (34.7)	170 (18.2)	1.75 (0.95–3.21)	0.08	1.76 (0.92–3.40)	0.09
Living situation, n (%)						
Living alone	24 (33.3)	278 (29.9)	1.13 (0.22–5.69)	0.89		
Living with partner/family	35 (48.6)	488 (52.5)	1.42 (0.24–8.53)	0.70		
Institution (rehabilitation centre or nursing	10 (13.8)	110 (11.8)		Ref.		
home)						
Number of medicines at discharge IH, mean (SD)	12.6 (5.4)	9.6 (5.7)	1.07 (1.04–1.12)	<0.01	1.02 (0.96–1.08)	0.57
Number of medication changes during IH, median + IQR	3 (2–6)	2 (1–4)	1.15 (1.03–1.29)	0.02	1.14 (1.05–1.24)	<0.01
0–1, n (%)	14 (19.4)	323 (34.7)				
2, n (%)	7 (9.7)	166 (17.8)				
3–4, n (%)	21 (29.2)	257 (27.6)				
≥5, n (%)	29 (40.3)	173 (18.6)				
eGFR<50 ml/min/1.73 m^2^, n (%)	24 (33.3)	166 (17.8)	1.32 (0.52–3.35)	0.56		
CCI score, median (IQR)	1 (0–3)	1 (0–2)	1.07 (0.95–1.21)	0.26		
0–1, n (%)	39 (54.1)	593 (63.7)				
2–3, n (%)	16 (22.2)	206 (21.8)				
≥4, n (%)	17 (23.6)	131 (14.1)				
**IH related**						
Unplanned IH, n (%)	60 (83.3)	751 (80.7)	1.02 (0.55–1.89)	0.94		
Duration of IH in days, median + IQR	7 (3–13)	5 (2–9)	1.01 (0.99–1.03)	0.43		
Time between IH and readmission in days, median + IQR	10.5 (4.3–18.9)	9 (4–17)	1.01 (0.97–1.06)	0.60		
Discharge on Saturday or Sunday, n (%)	11 (15.2)	143 (15.4)	1.03 (0.44–2.37)	0.95		
Planned post-discharge outpatient visit, n (%)	61 (84.7)	792 (85.2)	1.66 (0.24–11.24)	0.61		
≥3 hospital hospitalizations 6 months before IH, n (%)	19 (26.4)	118 (12.7)	1.66 (0.94–2.95)	0.08	2.11 (1.12–3.98)	0.02
≥2 ED visits (without a hospitalization),6 months before IH, n (%)	13 (18.1)	70 (7.5)	2.01 (1.05–3.86)	0.04	2.15 (1.00–4.65)	0.05
Discharge letter send to GP after discharge IH, n (%)	61 (84.7)	761 (81.8)	1.67 (0.83–3.34)	0.15		
Discharge letter send to GP ≤2 days after discharge IH, n (%)	22 (36.1)	222 (29.2)	1.52 (0.94–2.45)	0.09	1.37 (0.82–2.31)	0.23
Department, n (%)						
Internal medicine	17 (23.6)	190 (20.4)	-	Ref.		
Pulmonology	11 (15.3)	189 (20.3)	0.59 (0.09–3.72)	0.57		
Cardiology	17 (23.6)	113 (12.2)	1.12 (0.67–17.49)	0.94		
Surgery	12 (16.7)	264 (28.4)	0.45 (0.05–4.02)	0.48		
Gastroenterology	13 (18.0)	125 (13.4)	0.52 (0.22–1.22)	0.13		
Neurology^b^	2 (2.8)	42 (4.5)	-	-		
Psychiatry^b^	0	7 (1)	-	-		

GP, general practitioner; IH, index hospitalization; IQR, interquartile range; ED, emergency department; eGFR, estimated glomerular filtration rate.

^a^ Data were missing for: living situation (5.1%) and eGFR<50 ml/min/1.73 m^2^ (0.8%).

^b^ Too few cases to include in this analysis.

The updated Charlson comorbidity score (Quan et al., 2011) was derived from the hospital data system. Renal function was calculated using the Chronic Kidney disease Epidemiology Collaboration (CKD-EPI) formula. A cut-off of 50 ml/min/1.73 m^2^ was chosen based on the Dutch guidelines for dose adjustments in renal impairment. The presence of a language barrier was determined based on notes from the resident or nurse in the electronic patient file (e.g., use of an interpreter, difficulties in asking the medical history). After completion of the data entry the data were visually checked with histograms and scatterplots to check extreme values and missing data.

### Main Outcome Measures

The prevalence of medication-related readmissions (defined as the number of medication-related readmissions divided by the total number of unplanned readmissions) and the percentage of potentially preventable medication-related readmissions (defined as the number of potentially preventable medication-related readmissions divided by the total number of medication-related readmissions) were the main outcome measures. Additionally, the following measures for potentially preventable medication-related readmissions were assessed: potential risk factors (Table 1), type of medication and medication error that were involved.

### Data Analysis

Statistical analysis was performed in SPSS version 22.0 (IBM SPSS, Chicago, IL, United States). Categorical variables are reported as percentages. Normally and non-normally distributed continuous variables are reported as mean with standard deviation (SD) and median with interquartile range (IQR), respectively. To identify potential risk factors of potentially preventable medication-related readmissions and to adjust for patients with multiple readmissions, a univariate generalized estimating equation (GEE) with logistic regression was performed. Parameters with a p-value < 0.10 in the univariate analysis were entered into a multivariable model (Steyenberg. 2009 Edition). P-values lower than 0.05 were considered statistically significant. We checked for presence of collinearity (*R* > 0.5), reported unadjusted odds ratios (OR), adjusted odds ratios (OR_adj_) and 95% confidence intervals (CI). No pairwise correlations were above 0.5.

## Results

### Prevalence and Preventability

**FIGURE 2:**
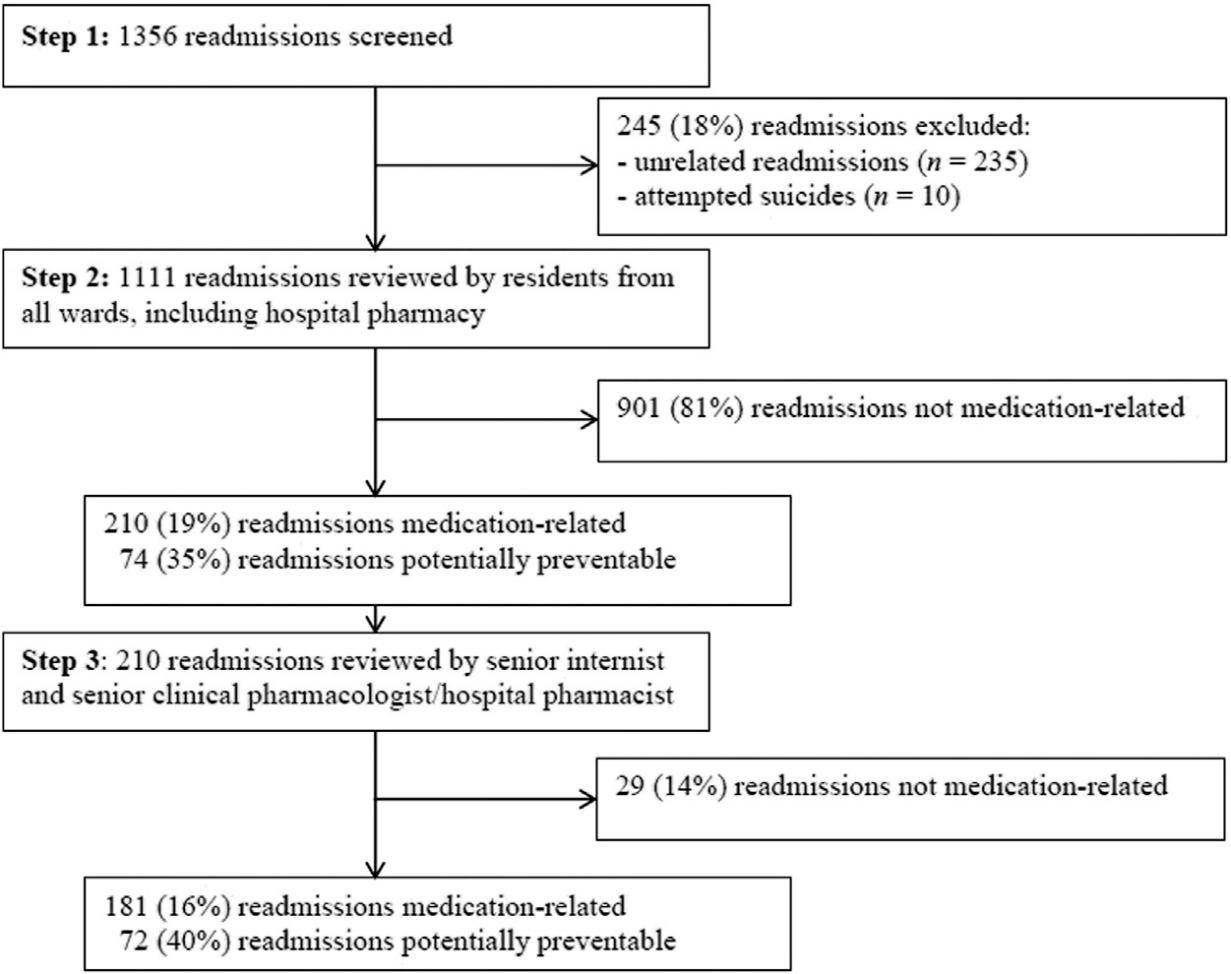
Study flow and main outcome.

Of the 1,356 readmissions screened, 245 (18%) were excluded (see Figure 2). This resulted in the inclusion of 1,111 readmissions for 873 unique patients: 699 patients had one readmission and 174 patients had two or more.

Initially, 210 readmissions were considered to be medication-related (step 2, Figure 1). After confirmation by a senior internist (C.S.) and senior clinical pharmacologist/hospital pharmacist (M.J.) (step 3, Figure 1), 181 of the 1,111 readmissions (16%) were considered medication-related, of which 72 (40%) were assessed as potentially preventable (Figure 2).

### Readmission Characteristics and Risk Factors

Table 1 shows the patient-related and IH-related characteristics of the potentially preventable medication-related readmissions (*n* = 72) vs. non-medication-related readmissions (*n* = 930). Mean age in the potentially preventable group was 69.5 years, 52.8% were men and the average number of medicines at discharge of the IH was 12.6.

Parameters independently associated with potentially preventable readmissions were number of medication changes during IH (ORadj: 1.14; 95% CI: 1.05–1.24) and three or more hospitalizations six months before IH (ORadj: 2.11; 95% CI: 1.12–3.98) (Table 1).

### Types of Medication and Reasons for Potentially Preventable Readmissions

The top five medications associated with potentially preventable medication-related readmissions were antidiabetics (15%), diuretics (15%), laxatives (14%), antithrombotic agents (10%) and medications for asthma/chronic obstructive pulmonary disease (COPD) (8%).

The most common reason for readmissions was cardiovascular symptoms (32%). Other reasons were poor glycemic control (15%), liver diseases (14%), gastrointestinal tract symptoms (6%), coagulation disorders (10%), respiratory symptoms (10%), central nervous system diseases (6%), electrolyte disturbances (6%) and infections (3%) (Table 2).

**TABLE 2:** Reasons for potentially preventable medication-related readmissions and the associated medication^a^.

Clinical presentation at readmission	Preventable readmissions (*n* = 72, 100%)	Associated medications (no. Of readmissions)
Circulatory		
Cardiovascular symptoms (e.g., heart failure, dysrhythmias, hyper- or hypotension)	23 (32%)	Diuretics (10), calcium antagonists (4), beta-blockers (3), medication affecting RAAS (3), cardiac glycosides (2), organic nitrates (1), theophylline (1), alpha-blocker (1)
Endocrine system		
Hypoglycemia or hyperglycemia	11 (15%)	Insulin (7), oral antidiabetics (3), corticosteroids (1)
Gastrointestinal system		
Hepatic encephalopathy/liver failure	10 (14%)	Laxatives (9), acetaminophen (1)
Gastrointestinal symptoms (diarrhea, constipation)	4 (6%)	Laxatives (1), loperamide (1), oral antidiabetics (1), antiemetics (1)
Blood		
Coagulation disorders (e.g., bleeding, anemia, embolism)	7 (10%)	Anticoagulants (7)
Respiratory system		
Respiratory symptoms (e.g., dyspnoea)	6 (8%)	Respiratory medication (5), opioids (1)
Central nervous system		
Epileptic seizure, pain, dysregulation of Parkinson's disease	4 (6%)	Opioids (1), antiepileptics (2), anti-Parkinson medication (1)
Electrolytes		
Electrolyte disturbance (e.g., hyper- or hypokalemia)	4 (6%)	Mineral supplements (2), medication for treatment of hyperkalemia (1), diuretics (1)
Immune system		
Infection	2 (3%)	Antibiotics (2)
Other		
Headache	1 (1%)	Infliximab (1)

^a^ More than one medicine per readmission is possible. RAAS, renin–angiotensin–aldosterone system.

### Medication Errors Involved in Potentially Preventable Readmissions

Table 3 shows the type of medication errors of the 72 potentially preventable medication-related readmissions. Prescribing errors (35%) and non-adherence (35%) were the most common medication errors. Underprescribing (40%), wrong dosage (24%) and inadequate monitoring (20%) were the most common sub-types of the prescribing errors.

**TABLE 3:** Type and subtype of medication errors involved in potentially preventable medication-related readmissions.

Type of error	Subtype of error	*n* (%)
Prescribing		25 (35)
	Underprescribing	10 (40)
	Dosage	6 (24)
	Inadequate monitoring	5 (20)
	No indication	3 (12)
	Contraindication	1 (4)
Across settings	Transition errors	22 (30)
Medication use	Non-adherence	25 (35)

## Discussion

This study shows that 16% of the readmissions are caused by medication and that 40% of these are potentially preventable. Most of the medication errors involved in the potentially preventable readmissions were classified as non-adherence (35%) and prescribing errors (35%), followed by transition errors (30%).

The frequency of 16% found in the current study is comparable to the median of 21% found in a systematic review of medication-related readmissions (El Morabet et al., 2018). Of the medication-related readmissions 40% were considered potentially preventable and the systematic review found that a median of 69% were preventable. The difference in preventability between studies could be caused by the case-mix of studies (e.g., including only geriatric patients vs. adult patients) and how preventability was assessed (e.g., monodisciplinary or multidisciplinary).

The finding that 40% of the medication-related readmissions were considered to be potentially preventable indicates that improvements may be possible. A total of 35% of the potentially preventable readmissions were due to prescribing errors, with underprescribing, wrong dosage and inadequate monitoring being the most common. To address underprescribing, a clinical medication review is needed to recognize the undertreated symptoms. To address inadequate monitoring, the clinical medication review should include a monitoring plan as well, describing when and how the effects of medication changes are evaluated and defining the responsibilities of the different care providers involved. Another 35% of the potentially preventable readmissions were due to medication non-adherence. Most of the previous studies investigating readmissions did not describe this relevant cause (Reynolds et al., 2014; El Morabet et al., 2018). Rosen et al. show that patients with low or intermediate medication adherence had more than 2.5-fold greater odds of being readmitted within 30 days (Rosen et al., 2017). However, non-adherence is a multifaceted problem (Kardas et al., 2013). The question is whether medication adherence is a modifiable predictor of hospital readmissions or a measure for underlying causes, such as socioeconomic, condition- or therapy-related factors, which are the true causes for readmission (Sabaté 2003). This is an important issue for future research. The final 30% of the potentially preventable readmissions were due to transition errors, including failure to communicate medication changes to the patient and/or the next healthcare providers. It remains a challenge to communicate medication changes after discharge, despite the efforts made in recent years to improve the transfer of medication-related information, including the implementation of medication reconciliation (Mueller et al., 2012). Interventions across the settings are needed, with specific recommendations to the patient and the next healthcare provider on what should be done after discharge. The study by Ravn-Nielsen et al. showed that a transitional pharmacist intervention, based on medication review, medication reconciliation, motivational interviews and follow-up after discharge, resulted in a significant reduction in the number of patients readmitted within 30 days (Ravn-Nielsen et al., 2018). The intensity of this intervention could explain its effectiveness. To implement such labor-intensive interventions in an effective way, a focus on patients at risk of adverse drug events is needed. The potential risk factors identified in this study might help to select these patients. A higher number of medication changes during IH and three or more hospital hospitalizations 6 months before IH were associated with potentially preventable medication-related readmissions within 30 days.

It would be interesting to explore the association between the clinical presentation during previous hospitalizations and this presentation during the preventable medication-related readmission. Possibly, adverse drug events were already present during the previous hospitalizations and index admission, but not recognized by the caregivers. As also described by Ravn-Nielsen et al. some Emergency Department visits or (re)admissions are not obviously medication-related (Ravn-Nielsen et al., 2018). If patients present themselves at the Emergency Department due to non-adherence, this will typically manifest itself as a worsening of his or her underlying disease and only if the patient indicates to be non-adherent this will be recognized as an adverse drug event. Apparently, in most cases the patient will be discharged without solving the adverse drug event, which could result in (re)admissions. Antidiabetics, diuretics, laxatives, antithrombotic agents and medications for asthma/COPD were most frequently involved. These agents correspond to medicines associated with medication-related admissions (Leendertse et al., 2008).

Although this is the first study to describe an extensive assessment of the prevalence and preventability of unplanned medication-related readmissions, it does have some limitations. First, this was a single-centre study and only the wards with the highest readmission rates in previous years were included, limiting the generalizability. However, reviewing files of readmitted patients is resource-intensive and so the selection of high-risk departments increased the focus of reviewers. Second, we used a two-step approach (resident review and confirmation by senior internist and hospital pharmacist) to assess the prevalence and preventability of medication-related readmissions. Some medication-related readmissions could have been missed if they were not recognized in the first step by the residents. However, our approach is likely to result in high specificity, which increases the reliability of the results. Third, during this study a TPC-program was implemented. As this program tends to improve the transfer of medication-related information to the patient and next healthcare providers, it is possible that it resulted in less medication related readmissions. Finally, the preventability of medication-related readmissions was assessed using information from medical records and adherence information based on the medication history. Relevant information from the patient or primary care providers not documented in the medical records could have been missed: for example, non-adherence due to practical problems or adverse drug reactions, leading to a higher preventability in reality. Furthermore, we examined non-adherence based on refill rates, which does not establish whether a patient actually uses his medication. However, the value of using refill rates in clinical research has been shown (Arnet et al., 2016).

In summary, this study shows that 16% of the readmissions are caused by medication, of which 40% are potentially preventable. If the results are confirmed in larger multicentre studies, this may indicate that more attention should be paid to medication-related harm as a cause of readmissions in order to lower the overall readmission rates.

## Data Availability

The raw data supporting the conclusions of this article will be made available by the authors, without undue reservation.
