# Supplementary material for: Medication-Related Hospital Readmissions Within 30 Days of Discharge: Prevalence, Preventability, Type of Medication Errors and Risk Factors
Source: Front Pharmacol. 2021 Apr 13;12:567424. doi: 10.3389/fphar.2021.567424 (PMC8077030; doi:10.3389/fphar.2021.567424)
Supplement: Supplementary file 1 [file datasheet1.docx]

Supplementary Material

**Table 1. Association between drug-related problems, adverse drug events, adverse drug reactions, and medication errors. Adapted from Otero and Schmitt (Otero et al. 2005, El Morabet et al 2018).**

|  | **Definition** | **Examples** |
| --- | --- | --- |
| **DRP** | Drug-related problem: an event or circumstance involving drug therapy that actually or potentially interferes with the desired health outcomes (Pharmaceutical Care Network Europe, 2019). | A patient with an adverse drug event, adverse drug reaction or medication error.  See examples below. |
| **ADE** | Adverse drug event: any injuries resulting from medication use, including physical harm, mental harm, or loss of function.  ADEs can result from adverse drug reactions (non-preventable) or medication errors (preventable) (Bates et al. 1995). | An allergic reaction in a patient with a penicillin allergy receiving amoxicillin (non-preventable if previously unknown), an allergic reaction in a patient with a known allergy receiving amoxicillin (preventable), a gastric bleeding in a patient with an NSAID an gastric ulcer in the history (preventable) |
| **ADR** | Adverse drug reaction: a response which is noxious and unintended, and which occurs at doses normally used in man for the prophylaxis, diagnosis, or therapy of disease, or for the modifications of physiological function (World Health Organization, 2006). | Antibiotic-induced diarrhea, cough due to an ACE inhibitor |
| **ME** | Medication error: errors in the process of prescribing, dispensing, or administering the medications that many cause or lead to inappropriate medication use or patient harm while the medication is the control of the health care professional, patient, or consumer (Leendertse et al. 2008, van den Bemt and Egberts 2007). | Administration of methotrexate daily instead of weekly causing patient harm (i.e. a preventable ADE). A wrong dose prescribed for a child but not causing harm either due to intercepting the error before reaching the child, or to the overdose not causing symptoms in the child (i.e. a potential ADE). |
| 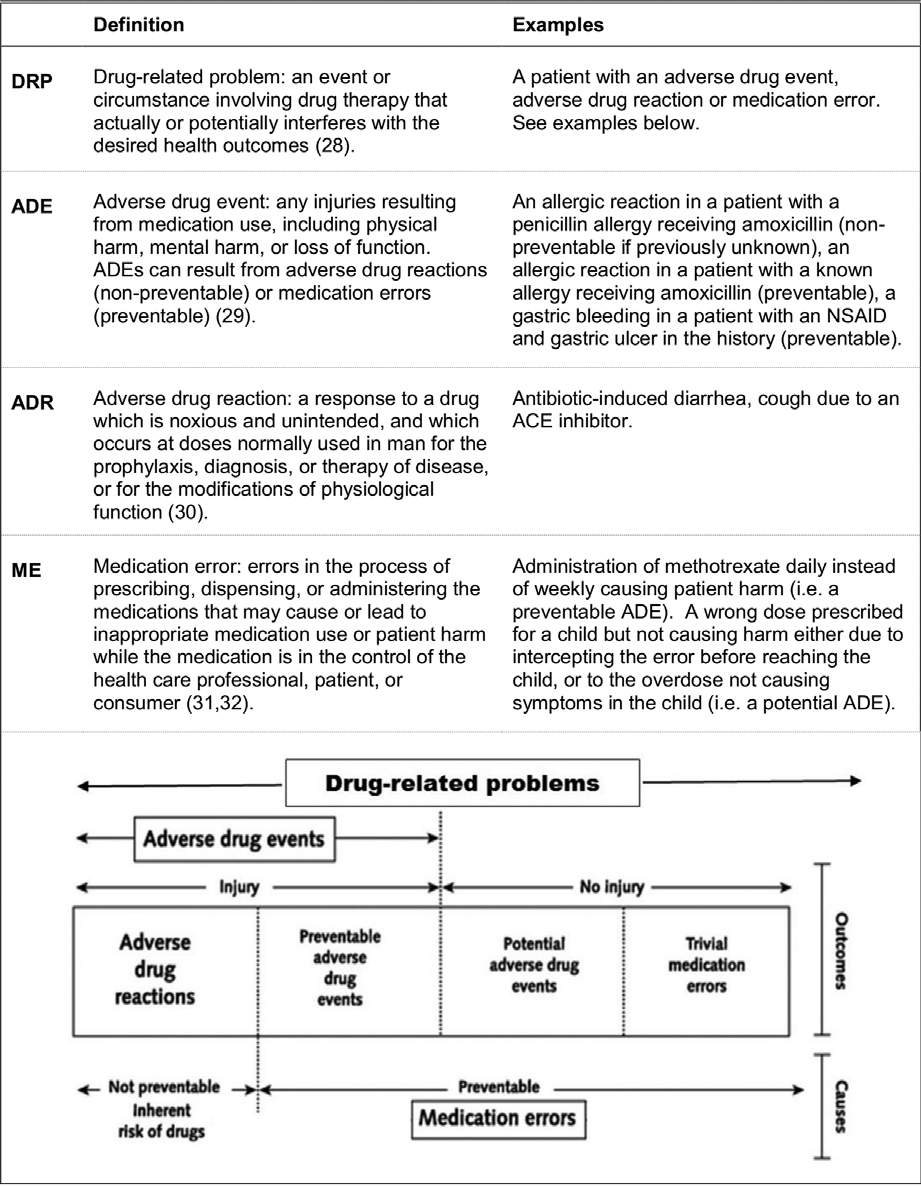 | | |

**Table 2: Causality assessment: i.e. whether the readmission is due to medication (Kramer et al. 1979, Leendertse et al. 2008)**

| **Question** |  |  |  |
| --- | --- | --- | --- |
| 1. Is the reason of (re)admission known to be an adverse event of the suspected medication? | Reason of (re)admission is known to be an adverse event of the medication  **Score:** +1 | Reason of (re)admission is unknown to be an adverse event of new medication (<5 year registered in Europe)  **Score:** 0 | Reason of (re)admission is unknown to be an adverse event of well known medication (>5 year registered in Europe)  **Score:** -1 |
| 2. Are there alternative causes that can explain the adverse event? | a) No alternative causes can explain the adverse event  **Score:** +2 | Alternative causes are present, but unlikely  **Score:** 0 | Possible other causes are present  **Score:** -1 |
|  | b) Inexplicable exacerbation or comeback underlying condition  **Score:** +1 |  |  |
| 3. Does a plausible time relationship exist between the adverse event and start of medication administration (or the occurrence of the  medication error)? | Time relationship as expected  **Score:** +1 | Time relationship is unclear  **Score:** 0 | Time relationship is not appropriate  **Score:** -2 |

The subscores of the three questions are added to a total score, and classified as following:

**Possible causal**: total score +4

**Probable causal :** total score 0 till 3

**Unlikely causal:** total score -4 tm -1

**Table 3 Preventability assessment: i.e. a medication error caused the readmission (Schumock and Thornton. 1992, McDonnell et al. 2002, Lau et al. 2003, Leendertse et al. 2008)**

| **Section A/ Section B**  Answering YES to one or more of the following implies that an ADE is potentially preventable.   1. Was there a history of allergy, previous reactions or contra-indication to the drug? 2. Was the drug involved inappropriate for the patient´s clinical condition (e,g. renal function, liver function, pregnancy)? 3. Was the dose, route, or frequency of administration inappropriate for the patient´s age, weight or disease state? 4. a. Was required therapeutic drug monitoring or other necessary laboratory tests not performed?  - b. If required therapeutic drug monitoring or other necessary laboratory tests were performed, insufficient actions has been taken?  1. Was a documented drug interaction involved in the ADE? 2. Was incorrect use of the drug involved in the ADE? (e.g. non-adherence) 3. a. Was a preventative measure not administrated to the patient?  - b. If a preventative measure was administrated, was it inadequate, and/or inappropriate?   If answers are all negative to the above, then proceed to Section C. |
| --- |
| **Section C**  The ADE is NOT preventable. |

**References**

Bates DW, Cullen DJ, Laird N, Petersen LA, Small SD, Servi D, et al. 1995. Incidence of adverse drug events and potential adverse drug events: implications for prevention. Jama.274(1):29-34.

Europe PCN. Classification for Drug related problems: The PCNE Classification V 6.2. Pharmaceutical Care Network Europe [on-line].

Kramer MS, Leventhal JM, Hutchinson TA, Feinstein AR 1979. An algorithm for the operational assessment of adverse drug reactions: Background, description, and instructions for use. Jama. 242:623-632.

Lau PM, Stewart K, Dooley MJ. 2003. Comment: hospital admissions resulting from preventable adverse drug reactions. Annals of Pharmacotherapy.37:303-304.

Leendertse AJ, Egberts AC, Stoker LJ, van den Bemt PM. 2008. Frequency of and risk factors for preventable medication-related hospital admissions in the Netherlands. Archives of internal medicine.168:1890-1896.

McDonnell PJ, Jacobs MR, 2002. Hospital admissions resulting from preventable adverse drug reactions. Annals of Pharmacotherapy. 36:1331-1336.

Otero M-J, Schmitt E, Nebeker JR, Samore MH, Barach P. 2005. Clarifying terminology for adverse drug events. Annals of internal medicine.142(1):77.

Schumock G, Thornton J. 1992: Focusing on the preventability of adverse drug reactions. Hospital pharmacy.27:538.

van den Bemt PM, Egberts A. 2007. Drug related problems: definitions and classification. EJHP Practice.2007:62-64.

World Health Organization 2006. Definitions. Accessed at https://www.who.int/medicines/areas/quality_safety/safety_efficacy/trainingcourses/definitions.pdf on 27 November 2019
